# Association between meeting 24‐h movement guidelines and academic performance in a sample of 67,281 Chinese children and adolescents

**DOI:** 10.1002/ejsc.12034

**Published:** 2024-03-18

**Authors:** Sitong Chen, Kaixin Liang, José Francisco López‐Gil, Clemens Drenowatz, Mark S. Tremblay

**Affiliations:** ^1^ Institute for Health and Sport Victoria University Melbourne Victoria Australia; ^2^ School of Psychology Shenzhen University Shenzhen China; ^3^ One Health Research Group Universidad de Las Américas Quito Ecuador; ^4^ Division of Sport Physical Activity and Health University of Education Upper Austria Linz Austria; ^5^ Children's Hospital of Eastern Ontario Research Institute and Department of Pediatrics University of Ottawa Ottawa Ontario Canada

**Keywords:** academic performance, physical activity, school‐aged student, screen time, sleep

## Abstract

This study aimed to explore the association between meeting 24‐h movement guidelines and academic performance among Chinese children and adolescents. Cross‐sectional data on 67,281 Chinese children and adolescents were used for the analyses. Adherence to 24‐h movement guidelines and academic performance (i.e., grades in Chinese, Math, and English), as well as sociodemographic information, were measured using a self‐reported questionnaire. 24‐h movement guidelines recommend at least 60 min/day of moderate to vigorous physical activity (MVPA), no more than 2 h/day of recreational screen time (ST), and 9–11 h/night of sleep for ages 11–13 years or 8–10 h/night for ages 14–17 years. Multilevel generalized linear models were used to explore the associations between 24‐h movement guidelines and academic performance. Results indicated that compared to not meeting any of the three 24‐h movement guidelines, participants meeting one or more guidelines were more likely to report better academic performance in Chinese, Math, and English. Meeting all the three guidelines was associated with better academic performance in Chinese (odds ratio [OR] = 1.56), Math (OR = 1.51), and English (OR = 1.73). Additionally, meeting the physical activity (PA) guidelines only was associated with better academic performance in Chinese; meeting only the ST guidelines, both ST and sleep guidelines, and both ST and PA guidelines were associated with better academic performance in Chinese, Math, and English. Subgroup analyses indicated that the associations between 24‐h movement guidelines and academic performance varied across grade groups and course subjects. As such, in general, adherence to 24‐h movement guidelines was related to better self‐reported academic performance. Healthy movement behaviors should be promoted for health and academic achievement in Chinese children and adolescents.

## Highlights

- Meeting 24‐h movement guidelines linked with better academic performance in Chinese children and adolescents.
- Adherence to the screen time (ST) guideline and any combination with the ST guideline showed the most consistent associations with better academic performance.
- Meeting the ST and physical activity guidelines concurrently showed the most favorable associations with academic performance.
- The association between 24‐h movement guidelines and academic performance varied across grade groups and course subjects.

## 1 INTRODUCTION

Healthy 24‐h movement behaviors (including physical activity [PA], sedentary behavior [SB], and sleep) are related to a variety of health benefits in children and adolescents. For example, movement behaviors at the recommended level have been found to be associated with various health outcomes (Saunders et al., 2016; Rollo et al., 2020), such as healthy weight management (Chen et al., 2021; García‐Hermoso et al., 2023; Roman‐Viñas et al., 2016), enhanced physical fitness (Chen et al., 2022; Tapia‐Serrano et al., 2023), reduced risks of depressive symptoms and anxiety (Liang et al., 2023; Sampasa‐Kanyinga et al., 2020), as well as healthful dietary patterns (Thivel et al., 2019; Yang et al., 2022). Such evidence supports the promotion of desired 24‐h movement behaviors on healthy growth and development in children and adolescents.

In the research field of healthy 24‐h movement behaviors, some researchers have started to explore the roles of the integrated behaviors on cognition‐related outcomes, such as academic performance (Wilhite et al., 2022). Academic performance refers to the degree of success in reaching educational goals, and it can be assessed by a series of indicators (Fan et al., 2001), such as exam scores, class grades, or subjective assessments. In China, academic performance is an important determinant of success. Owing to the traditional culture of this country (Chao, 1994), school‐aged students' academic performance has been a priority for families and schools (Li et al., 2018). Accordingly, Chinese children and adolescents are expected to pursue excellent academic performance for a bright future and positive development (Wu et al., 2019; Zhou et al., 2014) owing to the Chinese traditional academy‐oriented culture. Moreover, adolescents' academic performance may predict their subsequent psychosocial functioning (Adelantado‐Renau et al., 2022; Sörberg Wallin et al., 2019), with similar findings reported in the Chinese context (Feng et al., 2022; Jieyi et al., 2022).

A plethora of evidence has demonstrated separate associations of PA (Poitras et al., 2016), SB (with screen time [ST] often used as a surrogate) (Adelantado‐Renau et al., 2019), and sleep (Chaput et al., 2016) with academic performance in children and adolescents. As PA, ST, and sleep are interrelated and codependent daily movement behaviors within a 24‐h period (Chaput et al., 2014; Pedisic, 2014; Pedišić et al., 2017), recent studies have suggested that PA (especially moderate to vigorous intensity), ST, and sleep should be studied using an integrative approach in behavioral epidemiology research (Tremblay et al., 2016a, 2016b). Considering the various combinations of 24‐h movement guidelines, the overall prevalence of children and adolescents meeting all of them is, however, generally very low (Tapia‐Serrano, Sevil‐Serrano, et al., 2022). Many studies have adopted the integrative approach (“the whole day matters”) to investigate the association between 24‐h movement behaviors and academic performance in children and adolescents (Lien et al., 2020; Tapia‐Serrano, García‐Hermoso, et al., 2022; Watson et al., 2022). For example, Lien et al. (2020) found that adolescents who met the 24‐h movement guidelines were more likely to self‐report better academic performance, but the authors only used a single question to assess the participants' perceived overall academic performance. Based on Australian data, Watson et al. (2022) found that children meeting all 24‐h movement guidelines had better literacy performance than those meeting none of the guidelines. Tapia‐Serrano, García‐Hermoso, et al. (2022) suggested that meeting two or more recommendations in the 24‐h movement guidelines was likely to be linked with better academic performance.

Based on these studies, it seems that the association between 24‐h movement guidelines and academic performance in children and adolescents needs more clarification. Due to the mixed results found, it seems necessary to conduct further studies to help clarify the association between 24‐h movement guidelines and academic performance because there is a lack of evidence based on Chinese samples. As Chinese society puts considerable emphasis on academic performance, a large part of children's and adolescents' time is spent on academic activities at the expense of time for PA and sleep (Chen et al., 2021). As such, the health benefits of sufficient PA (Poitras et al., 2016) and appropriate sleep (Chaput et al., 2016) are prone to be neglected. If studies identify a favorable association between 24‐h movement behaviors and academic performance, findings can inform policymakers to formulate and implement policies that are more conducive to children's and adolescents' healthy development as well as academic performance. Further, only a few studies focused on overall academic performance, rather than examining the association between movement guidelines and academic performance across different course subjects. Considering that the characteristics differ between course subjects (e.g., Chinese subject is more concerned with reading comprehension, while Math may require more advanced logical abilities), the association between 24‐h movement behaviors and academic performance may be different across course subjects.

Therefore, this study aims to advance the current evidence base concerning the association between meeting the 24‐h movement guidelines and academic performance in a large sample of Chinese children and adolescents.

## 2 MATERIALS AND METHODS

### 2.1 Participants and study design

In collaboration with the Municipal Education Commission, a large‐scale survey was conducted in primary and middle schools in Shenzhen, China, in March 2021. The sample included students from local public primary and secondary schools from all districts of Shenzhen. Previous PA related studies in Chinese children and adolescents suggested that children at 10 years or older had sufficient reading comprehension and the ability to complete the self‐report questionnaire (Chen et al., 2021), and also the Municipal Education Commission recommended to conduct survey in school‐going students of grades 5 and above. Consequently, students in grades 5 and above were considered as eligible study participants. Accordingly, the study included students in primary school, junior middle school, and high school.

Participants and their guardians were informed of the study's aim through a written document. This survey is intended to collect data about physical and mental outcomes, movement behaviors, and sociodemographic variables. Each student spent about 20 min completing the survey, voluntarily, and data were collected anonymously. The students willing to participate completed the online questionnaire independently in the school computer room during a class period with a teacher's presence to assist with the survey. The questionnaire was completed on a Chinese online questionnaire platform (https://www.wjx.cn/). Before entering the formal response page, the electronic informed consent page was displayed to the participants. Only those who agreed to participate in the survey would further enter the pages for formal data collection. The survey was approved by the University Research Committee (No. 2020005) and participating schools.

**FIGURE 1:**
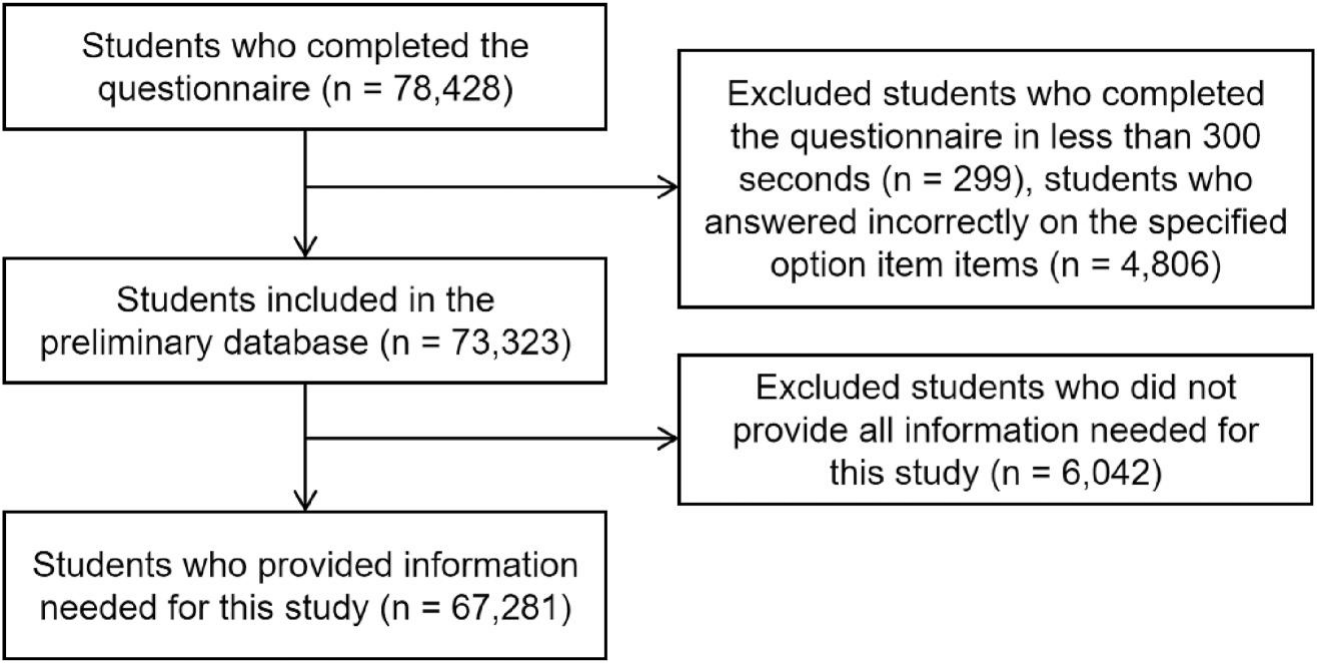
Detailed process used for cleaning invalid and missing data.

A total of 78,428 participants completed the questionnaire. After deleting the data of participants who completed the questionnaire in less than 300 s and answered incorrectly on the specified option item and considering the availability of variables required for the current study, the sample for the analyses included 67,281 students. Details about cleaning invalid and missing data in this study are presented in Figure 1.

### 2.2 Measures

Sleep time was measured using the Pittsburgh sleep quality index (PSQI) entry on sleep time: “How many hours did you actually sleep at night in the past month?” The Chinese version of the PSQI has been verified among Chinese adolescents (Ho et al., 2021). ST was assessed using the relevant items in the Health Behavior of School‐aged Children (HBSC) survey, including the time spent on various screen activities (i.e., TV/movies, video games, and other screen‐based activities during leisure time) in the past 7 days. The average daily ST was calculated by the following formula: ([sum of ST on weekdays × 5] + [sum of weekend day ST × 2])/7. PA was assessed by one question adapted from the HBSC survey, that was “How many days did you engage in moderate to vigorous physical activity (MVPA) at least 60 min on weekdays over the past week? (0 = none, 1 = 1 day, 2 = 2 days, 3 = 3 days, 4 = 4 days, 5 = 5 days, 6 = 6 days, and 7 = 7 days)”. This item has been shown satisfactory reliability among Chinese adolescents (Liu et al., 2010) and has been used widely in Chinese children and adolescents (Chen et al., 2020, 2021, 2023; Liang et al., 2023). According to the Canadian 24‐Hour Movement Guidelines for Children and Youth (Tremblay et al., 2016a), reporting 7 days with at least 60 min of MVPA was considered as meeting the PA guidelines; no more than two hours of ST per day was considered as meeting the ST guidelines; having 9–11 h and 8–10 h of sleep for children (5–12 years) and adolescents (13–17 years), respectively, was considered as meeting the sleep guidelines. The combined cases of meeting the three guidelines can be determined as either the number of guidelines met (i.e., none, one, two, or three) or specific combinations (i.e., none, PA only, ST only, sleep only, MVPA + ST, MVPA + sleep, ST + sleep, MVPA + ST + sleep).

Academic performance for Chinese, Math, and English was self‐reported by study participants as grades of each subject in the last final exam of the previous semester. In China, the examination results of students in the compulsory education stage are presented as grades. Specifically, students' grades range from A to F (downwards), with A indicating the highest while F indicating the lowest grade. The Youth Risk Behavior Survey also uses similar questions and responses to measure participants' academic performance (Brener et al., 2013).

Additionally, the following information was gathered: sex (male/female), grade (i.e., primary school/junior middle school/senior middle school), self‐reported height (cm) and weight (kg), number of siblings (i.e., only one child/not only child), family structure (both parents/single), parental education level (i.e., junior middle school or below/high school or equivalent/bachelor or equivalent/master or above/unclear), ethnicity (i.e., Han/minority), family socioeconomic status (SES), district (nine districts in total), and school (135 schools in total). Age‐ and sex‐specific weight status based on weight and height was determined from the body mass index (BMI, calculated by self‐reported height and weight) according to China's norm‐referenced data (Zhu et al., 2019), classifying participants into one of three categories (normal weight, overweight, and obese). SES was measured using the adapted version of the MacArthur Scale of Subjective Social Status (a 10‐rung ladder with higher scores indicating better SES) (Cundiff et al., 2013). In the previous studies, those variables were controlled for in the statistical analyses to minimize the confounding bias (Lien et al., 2020; Marciano et al., 2021a; Tapia‐Serrano, García‐Hermoso, et al., 2022; Watson et al., 2022).

### 2.3 Data analysis

After removing participants with invalid or incomplete data (*n* = 11,147), 67,281 participants were included as the analytical sample. All statistical analyses were performed using STATA BE 17.0 (College Station, Texas, USA). Descriptive statistics were used to report sample characteristics. Based on the data structure and the fact that data from different layers were nested, a three‐level mixed multilevel effect model (level 3: district; level 2: school; and level 1: individual; such layers were based on sampling strategy) was used to assess the associations of meeting the 24‐h movement guidelines and academic performance indicators (Chinese, Math, and English). According to the essence of the dependent outcome (academic performance), it was treated as an ordinal variable in further analysis. Therefore, a series of ordinal models were developed to assess the associations between meeting the 24‐h movement guidelines and academic performance while controlling all the sociodemographic variables mentioned above (e.g., SES and sex). In detail, model 1 (number of recommendations met; “none” as the reference group) and model 2 (specific combinations met; “none” as the reference group) focused on the associations between meeting 24‐h movement guidelines and Chinese (“grade F” as the reference group); model 3 (number of recommendations met; “none” as the reference group) and model 4 (specific combinations met; none as the reference group) focused on associations between meeting 24‐h movement guidelines and Math (“grade F” as the reference group); model 5 (number of recommendations met; “none” as the reference group) and model 6 (specific combinations met; “none” as the reference group) focused on associations between meeting 24‐h movement guidelines and English (“grade F” as reference group). An interaction effect of the grade group and 24‐h movement guidelines on academic performance was observed; thus, analyses by grade groups were conducted while controlling the sociodemographic variables except for the grade group. Results are presented as odds ratio (OR) with a 95% confidence interval (CI). A *p*‐value below 0.05 was used for statistical significance.

## 3 RESULTS

The sociodemographic characteristics of study participants are shown in Table 1. A total of 19.6% of participants met the sleep guideline, 61.0% of participants met the ST guideline, and 9.3% of participants met the MVPA guideline. Overall, 1.7% of participants met all three guidelines while 28.7% of participants did not meet any.

**TABLE 1:** Descriptive characteristics of participants.

Categorical variables		*n*	%
Sex	Boys	34,909	51.9
	Girls	32,372	48.1
Grade	Primary school	27,954	41.5
	Junior middle school	27,124	40.3
	High school	12,203	18.1
Weight status	Normal	45,817	68.1
	Overweight	9051	13.5
	Obese	12,413	18.4
Siblings	Only child	17,354	25.8
	Not only child	49,927	74.2
Family structure	Both parents	62,836	93.4
	Single	4445	6.6
Paternal education	Junior middle school or below	14,619	21.7
	High school or equivalent	18,159	27
	Bachelor or equivalent	26,030	38.7
	Master or above	2796	4.2
	Unclear	5677	8.4
Maternal education	Junior middle school or below	17,617	26.2
	High school or equivalent	18,706	27.8
	Bachelor or equivalent	23,922	35.6
	Master or above	1635	2.4
	Unclear	5401	8
Nationality	Han	65,027	96.6
	Minority	2254	3.4
Number of guidelines met	0	19,277	28.7
	1	36,712	54.6
	2	10,174	15.1
	3	1118	1.7
Specific combinations of guidelines met	None	19,277	28.7
	Sleep only	4611	6.9
	Screen time only	30,336	45.1
	Physical activity only	1765	2.6
	Sleep and screen time	6823	10.1
	Sleep and physical activity	603	0.9
	Screen time and physical activity	2748	4.1
	All three	1118	1.7
Chinese grades	A	25,032	37.2
	B	28,257	42
	C	10,625	15.8
	D	2624	3.9
	E	415	0.6
	F	328	0.5
Math grades	A	22,842	34
	B	22,868	34
	C	13,631	20.3
	D	5832	8.7
	E	1295	1.9
	F	813	1.2
English grades	A	22,153	32.9
	B	22,331	33.2
	C	14,091	20.9
	D	6435	9.6
	E	1409	2.1
	F	862	1.3

Continuous variables	Mean	SD
Age (years)	13	1.8
Subjective family socioeconomic status	5	1.7

*Note*: Subjective family socioeconomic status was scaled from 0 to 10.

Abbreviation: SD, standard deviation.

Associations between meeting 24‐h movement guidelines and academic performance in Chinese are shown in Table 2. Participants meeting one or more guidelines were more likely to report better grades in Chinese (one guideline: OR = 1.41 [1.36, 1.45], *p* < 0.001; two guidelines: OR = 1.52 [1.45, 1.59], *p* < 0.001; and three guidelines: OR = 1.59 [1.41, 1.80], *p* < 0.001). Regarding specific combinations of guidelines, meeting only the ST recommendation (OR = 1.52 [1.46, 1.57], *p* < 0.001), only the MVPA recommendation (OR = 1.12 [1.02, 1.23], *p* = 0.016), sleep + ST recommendations (OR = 1.49 [1.41, 1.57], *p* < 0.001), ST + MVPA recommendation (OR = 1.67 [1.55, 1.81], *p* < 0.001), and all three recommendations (OR = 1.56 [1.39, 1.76], *p* < 0.001) were significantly associated with higher grades in Chinese.

**TABLE 2:** Associations between meeting 24‐h movement guidelines with academic performance.

	Chinese			Math			English		
	OR	95% CI	*p*	OR	95% CI	*p*	OR	95% CI	*p*
Number of guidelines met									
0	Reference			Reference			Reference		
1	1.41	1.36, 1.45	<0.001	1.45	1.40, 1.50	<0.001	1.47	1.42, 1.52	<0.001
2	1.52	1.45, 1.59	<0.001	1.53	1.46, 1.61	<0.001	1.56	1.49, 1.63	<0.001
3	1.59	1.41, 1.80	<0.001	1.54	1.37, 1.73	<0.001	1.77	1.58, 1.99	<0.001
Specific guidelines met									
None	Reference			Reference			Reference		
Sleep only	0.98	0.93, 1.05	0.619	1.00	0.95, 1.07	0.902	0.96	0.90, 1.02	0.173
Screen time only	1.52	1.46, 1.57	<0.001	1.58	1.53, 1.64	<0.001	1.62	1.56, 1.67	<0.001
Physical activity only	1.12	1.02, 1.23	0.016	0.93	0.85, 1.02	0.143	1.02	0.93, 1.11	0.745
Sleep and screen time	1.49	1.41, 1.57	<0.001	1.50	1.43, 1.59	<0.001	1.55	1.47, 1.63	<0.001
Sleep and physical activity	1.08	0.93, 1.26	0.318	1.05	0.90, 1.22	0.532	1.05	0.91, 1.22	0.503
Screen time and physical activity	1.67	1.55, 1.81	<0.001	1.68	1.55, 1.81	<0.001	1.64	1.52, 1.77	<0.001
All three	1.56	1.39, 1.76	<0.001	1.51	1.34, 1.70	<0.001	1.73	1.54, 1.95	<0.001

*Note*: All models controlled all the sociodemographic variables.

Abbreviations: CI, confidence interval; OR, odds ratio.

Table 2 presents associations between meeting 24‐h movement guidelines and academic performance in Math. Participants who met one or more guidelines were more likely to obtain greater grades (one guideline: OR = 1.45 [1.40, 1.50], *p* < 0.001; two guidelines: OR = 1.53 [1.46, 1.61], *p* < 0.001; and three guidelines: OR = 1.54 [1.37, 1.73], *p* < 0.001). Specifically, meeting only the ST guideline (OR = 1.58 [1.53, 1.54], *p* < 0.001), sleep + ST guidelines (OR = 1.50 [1.43, 1.59], *p* < 0.001), ST + PA guidelines (OR = 1.68 [1.55, 1.81], *p* < 0.001), and all three guidelines (OR = 1.51 [1.34, 1.70], *p* < 0.001) were associated with higher odds of greater grades in Math.

Table 2 also presents associations between meeting 24‐h movement guidelines and academic performance in English. Meeting one or more guidelines was associated with higher odds of reporting higher grades in English (one guideline: OR = 1.47 [1.42, 1.52], *p* < 0.001; two guidelines: OR = 1.56 [1.49, 1.63], *p* < 0.001; and three guidelines: OR = 1.77 [1.58, 1.99], *p* < 0.001). Meeting only the ST guideline (OR = 1.62 [1.56, 1.67], *p* < 0.001), sleep + ST guidelines (OR = 1.55 [1.47, 1.63], *p* < 0.001), ST + PA guidelines (OR = 1.64 [1.52, 1.77], *p* < 0.001), and all three guidelines (OR = 1.73 [1.54, 1.95], *p* < 0.001) were associated with higher odds of higher grades in English.

The associations between meeting the 24‐h guidelines and academic performance of course subjects by the grade group are shown in Table 3. In terms of the number of guidelines met and academic performance in course subjects, it was found that meeting more guidelines was incrementally and favorably associated with better academic performance across all the course subjects in primary school students. In junior middle school students, meeting more guidelines was favorably associated with better academic performance in all the course subjects but not in a manner of dose–response relationship. Regardless of course subjects, in high school students, meeting all the three 24‐h movement guidelines was not associated with academic performance.

**TABLE 3:** Associations between meeting 24‐h movement guidelines and academic performance by grade.

	Chinese								
	Primary school			Middle school			High school		
	OR	95% CI	*p*	OR	95% CI	*p*	OR	95% CI	*p*
Number of guidelines met									
0	Reference			Reference			Reference		
1	1.49	1.41, 1.58	<0.001	1.48	1.41, 1.55	<0.001	1.16	1.08, 1.25	<0.001
2	1.70	1.58, 1.82	<0.001	1.51	1.40, 1.64	<0.001	1.17	1.02, 1.35	0.030
3	1.84	1.59, 2.12	<0.001	1.37	1.09, 1.73	0.007	1.54	0.76, 3.11	0.228
Specific guidelines met									
None	Reference			Reference			Reference		
Sleep only	1.16	1.07, 1.27	0.001	0.88	0.79, 0.98	0.015	0.83	0.69, 1.01	0.061
Screen time only	1.63	1.54, 1.74	<0.001	1.62	1.53, 1.70	<0.001	1.19	1.10, 1.28	<0.001
Physical activity only	1.15	1.00, 1.34	0.057	1.17	1.03, 1.34	0.015	1.08	0.77, 1.51	0.666
Sleep and screen time	1.67	1.55, 1.80	<0.001	1.48	1.34, 1.63	<0.001	1.18	1.01, 1.39	0.040
Sleep and physical activity	1.30	1.07, 1.58	0.010	0.84	0.64, 1.10	0.200	1.36	0.70, 2.65	0.371
Screen time and physical activity	1.95	1.73, 2.20	<0.001	1.67	1.49, 1.86	<0.001	1.09	0.83, 1.43	0.546
All three	1.83	1.58, 2.12	<0.001	1.35	1.07, 1.71	0.011	1.52	0.75, 3.07	0.240

	Math									
	Primary school				Middle school			High school		
	OR	95% CI		*p*	OR	95% CI	*p*	OR	95% CI	*p*
Number of guidelines met										
0	Reference				Reference			Reference		
1	1.53	1.45, 1.62		<0.001	1.59	1.52, 1.67	<0.001	1.10	1.03, 1.19	0.007
2	1.66	1.56, 1.78		<0.001	1.67	1.55, 1.80	<0.001	1.17	1.02, 1.35	0.021
3	1.81	1.57, 2.09		<0.001	1.42	1.13, 1.78	0.003	1.03	0.50, 2.14	0.930
Specific guidelines met										
None	Reference				Reference			Reference		
Sleep only	1.14	1.04, 1.24		0.003	0.94	0.85, 1.04	0.236	1.18	0.98, 1.42	0.084
Screen time only	1.73	1.63, 1.83		<0.001	1.78	1.69, 1.88	<0.001	1.10	1.02, 1.18	0.010
Physical activity only	0.99	0.86	1.14	0.907	0.96	0.84, 1.08	0.480	1.03	0.74, 1.42	0.865
Sleep and screen time	1.65	1.54	1.78	<0.001	1.71	1.55, 1.88	<0.001	1.15	0.99, 1.35	0.072
Sleep and physical activity	1.13	0.93, 1.37		0.224	1.04	0.79, 1.36	0.798	1.86	1.00, 3.46	0.051
Screen time and physical activity	1.94	1.72, 2.18		<0.001	1.70	1.53, 1.90	<0.001	1.16	0.88, 1.51	0.294
All three	1.80	1.56, 2.08		<0.001	1.39	1.11, 1.75	0.004	1.04	0.50, 2.14	0.921

	English								
	Primary school			Middle school			High school		
	OR	95% CI	*p*	OR	95% CI	*p*	OR	95% CI	*p*
Number of guidelines met									
0	Reference			Reference			Reference		
1	1.53	1.45, 1.62	<0.001	1.60	1.53, 1.68	<0.001	1.11	1.04, 1.20	0.003
2	1.73	1.62, 1.85	<0.001	1.53	1.42, 1.65	<0.001	1.24	1.08, 1.43	0.002
3	2.04	1.76, 2.36	<0.001	1.55	1.24, 1.93	<0.001	1.81	0.91, 3.63	0.092
Specific guidelines met									
None	Reference			Reference			Reference		
Sleep only	1.09	1.00, 1.19	0.041	0.93	0.84, 1.03	0.171	0.87	0.72,1.04	0.135
Screen time only	1.74	1.64, 1.84	<0.001	1.78	1.70, 1.88	<0.001	1.14	1.06, 1.22	0.001
Physical activity only	1.10	0.96, 1.27	0.182	1.07	0.94, 1.21	0.309	0.91	0.66, 1.26	0.578
Sleep and screen time	1.72	1.59, 1.85	<0.001	1.57	1.43, 1.73	<0.001	1.26	1.08, 1.48	0.004
Sleep and physical activity	1.20	0.99, 1.45	0.061	0.86	0.66, 1.12	0.261	2.28	1.18, 4.42	0.014
Screen time and physical activity	2.02	1.79, 2.27	<0.001	1.57	1.41, 1.75	<0.001	1.07	0.82, 1.40	0.624
All three	2.03	1.76, 2.35	<0.001	1.52	1.22, 1.90	<0.001	1.80	0.90, 3.60	0.096

*Note*: All models controlled all the sociodemographic variables except for the grade group.

Abbreviations: CI, confidence interval; OR, odds ratio.

For Chinese of primary school students, any combinations other than “meeting the PA guidelines only” were associated with better academic performance. For Chinese of junior middle school students, any combinations other than “meeting the Sleep + PA guidelines” were associated with better academic performance. For Chinese of high school students, combinations of “meeting the Sleep guidelines only” and “meeting the PA guidelines only” were associated with better academic performance. For Math of primary school students, any combinations except for “meeting the PA guidelines only” and “meeting the Sleep + PA guidelines” were associated with better academic performance. For Math of junior middle school students, any combinations except for “meeting the Sleep guidelines only”, “meeting the PA guidelines only”, and “meeting the Sleep + PA guidelines” were associated with better academic performance. For Math of high school students, meeting only the ST guideline was associated with enhanced academic performance. For English of primary school students, any combinations except for “meeting the PA guidelines only” and “meeting the Sleep + PA guidelines” were associated with better academic performance. For English of junior middle school students, any combinations except for “meeting the Sleep only guidelines”, “meeting the PA guidelines only”, and “meeting the Sleep + PA guidelines” were associated with better academic performance. For English of high school students, combinations of “meeting the Sleep guidelines only”, “meeting the PA guidelines only”, and “meeting the Sleep + PA guidelines” were positively associated academic performance. More details can be found in Table 3.

## 4 DISCUSSION

This study aimed to explore the association between meeting the 24‐h movement guidelines and academic performance. The current study found the following: (1) meeting all three 24‐h movement guidelines was associated with higher academic performance; (2) meeting more recommendations of the 24‐h movement guidelines was associated with better academic performance compared to meeting fewer ones; (3) meeting both the MVPA and ST guidelines was likely to show the strongest positive association with academic performances; and (4) the associations between 24‐h movement guidelines and academic performance in different course subjects varied across grade groups.

Our results are consistent with previous studies (Lien et al., 2020; Marciano et al., 2021a) that meeting all the recommendations would positively associate with academic performance. For example, a study consisting of Swiss students suggested that meeting three 24‐h movement guidelines was prospectively associated with better academic performance (Marciano et al., 2021a). Similarly, a cross‐sectional study consisting of Canadian adolescents found that meeting all the 24‐h movement guidelines was more likely to report better academic performance in adolescents (Lien et al., 2020). Concerning the underlying mechanism, studies have shown that memory, attention span, and executive functions as well as brain structures can be benefited by either sufficient PA (Vazou et al., 2019; Álvarez‐Bueno et al., 2017), limited ST (Marciano, Camerini, et al., 2021) and adequate sleep duration (Cheng et al., 2021; Kuhn et al., 2016). The improvements of these functions could subsequently enhance academic performance in children and adolescents. Moreover, it has been demonstrated that ST has positive effects on memory and cognition. Furthermore, evidence has indicated that meeting all three 24‐h movement guidelines might improve cognitive function in children and adolescents (Draper et al., 2020; Walsh et al., 2018; Zeng et al., 2022), which could further enhance academic performance (Duncan et al., 2007).

Compared with meeting none of the 24‐h movement guidelines, meeting more recommendations is more likely to be associated with better academic performance, in a dose–response manner (i.e., 3 > 2 > 1 > 0). To our knowledge, very few studies have found such a dose–response association because most previous studies focused on the specific combinations of guidelines met and their association with academic performance. In this regard, our study can support the key health message that either sufficient MVPA, limited recreational ST, or sufficient sleep is good, but including all of these behaviors would lead to greater benefits in terms of academic performance among children and adolescents (Tremblay et al., 2016a). This finding is expected because previous studies have suggested that the recommended levels of MVPA, ST, and sleep are independent contributors to improved academic performance, and thus, theoretically, combinations should lead to an incrementally beneficial result. However, the dose–response association in our study is simply based on our observed results, which needs more prospective or experimental evidence to understand the casual association between 24‐h movement guidelines and academic performance.

A previous systematic review with meta‐analysis found a low prevalence of children and adolescents meeting all three recommendations (Tapia‐Serrano, Sevil‐Serrano, et al., 2022), which is consistent with our study (e.g., 1.7% of the sample in this study). In this regard, enhancing academic performance by promoting meeting all the 24‐h movement guidelines is challenging to achieve. Thus, identifying which specific combination(s) within the 24‐h movement guidelines are most beneficial may be a more feasible approach. In this study, we found that regardless of course subjects, meeting the ST guideline individually, or meeting the ST guideline in combination with either PA or sleep guidelines, showed higher odds for better academic performance than any other combination(s). Particularly, of all the subjects examined, meeting the PA (except for Chinese) or sleep guidelines individually was not associated with better academic performance. Overall, these results highlight the potential role of limiting ST in promoting academic performance in Chinese children and adolescents. However, it is acknowledged that the underlying mechanisms were not addressed in this study.

When looking at the associations meeting between different combinations within the 24‐h movement guidelines and academic performance in different course subjects, it was only found that the association between meeting the PA guidelines individually and academic performance was inconsistent, while the other associations were consistent. Specifically, meeting the PA guidelines was only positively associated with academic performance in Chinese. There has been evidence supporting that meeting the PA guidelines is not associated with academic performance in Math or English (Howie et al., 2020) because specific forms of PA matter. Therefore, the differences in the associations of meeting the PA guidelines with academic performance in different course subjects may be due to forms of PA as evidence has confirmed associations between the specific forms of PA and academic performance (Howie et al., 2012, 2020). In our study, the measure to PA was board, which cannot provide detailed information on different modalities of PA. Considering the unique characteristics in learning different course subjects, different cognitive functions may be required (Lubans et al., 2016). For example, Chinese learning needs more comprehensions of abstract concepts (Chen and Altarriba, 1993), while Math learning needs advanced logical reasoning (Greenes, 2008). These explanations could be useful in better understanding the associations between meeting the PA guidelines and academic performance in different course subjects but require further mechanistic analyses.

Concerning the associations between 24‐h movement guidelines and academic performance in different course subjects, large variations across grade groups were observed. Specifically, in terms of the number of guidelines met, it was found that meeting all the 24‐h movement guidelines was not associated with any course subjects' academic performance in high school students. Moreover, for the specific combinations, more inconsistent results were found. For example, meeting the sleep guidelines individually was significantly associated with academic performance in Chinese in primary and middle school students but not high school students; for Math, nearly all the combinations of 24‐h movement guidelines were not associated with academic performance in high school students, but in other grade groups. The apparent discrepancy could be explained by the following possible reasons. First, in high school students, improvements in academic performance in Chinese high school students are primarily determined by academic assignments. This is because in China, the goal of high school learning is to purse high‐quality university education through the national college entrance examination, in which high school students have to complete a large number of academic assignments to purse the goal. In this regard, healthy movement behaviors may a low association. This can also explain why the dose–response relationship between the number of guidelines and academic performance was only significant in primary school students as they are not suffering from heavy academic pressures. Second, owing to onerous time for heavy academic workloads, high school students have little opportunities to engage in sufficient PA and have adequate sleep duration (Chen et al., 2021), which could in part explain why meeting the PA or sleep guidelines was not associated with academic performance regardless of course subjects. However, the above assumptions need more thorough explorations.

This is one of the very few studies assessing the association between 24‐h movement guidelines and academic performance in children and adolescents and extends the scientific evidence on the benefits of optimal 24‐h movement behaviors in a Chinese population. It is beneficial to provide an evidence base for Chinese stakeholders (e.g., school and family) to encourage kids to ‘move more, sit less, and sleep well’, but also to provide information for the refinement of 24‐h movement guidelines regarding the promotion of academic performance across different countries. Some practical implications can still be drawn. First, promoting optimal 24‐h movement behaviors is strongly recommended for promoting academic performance in Chinese children and adolescents. Second, as meeting all three recommendations of the 24‐h movement guidelines appears a challenge, meeting as many recommendations as possible is desirable. Third, if meeting all three 24‐h movement guidelines is a challenge, considering limiting ST and increasing MVPA opportunities in school settings as MVPA and ST are viable while sleep time could not be directly affected in school settings. Fourth, age‐specific strategies aimed at improving 24‐h movement behaviors should be considered and implemented.

This study has some strengths; first it included a large sample size for enhanced research generalizability. Second, many covariates were included in the statistical analysis, thereby increasing the quality of the findings. Nevertheless, some study limitations have to be mentioned. Owing to the cross‐sectional study design, it is not possible to infer causal associations. More improved studies are needed to reveal the casual association between 24‐h movement guidelines and academic performance in children and adolescents, and future studies should seek to elucidate the underlying mechanism(s) linking the 24‐h movement guidelines and academic performance. Self‐reported questionnaires were used to collect data on all the variables, which is subject to study participants' recall and social desirability biases. Therefore, some measurement issues (e.g., not capturing all kinds of recreational ST) should be considered when interpreting study findings. Furthermore, some important confounding factors (e.g., parenting styles) were not included in our study, which may influence our study results.

## 5 CONCLUSION

Meeting more recommendations in the 24‐h movement guidelines was incrementally and positively associated with better academic performance in Chinese children and adolescents. Ideally, encouraging children and adolescents to meet all three recommendations could generally show the most favorable associations with academic performance. However, if it is challenging to meet all three recommendations, it is recommended to promote PA while limiting ST to improve academic performance, especially for primary and middle school students. Given that this study was based on one Chinese city, it should be cautious to extrapolate study findings into wider contexts.

## CONFLICT OF INTEREST STATEMENT

The authors report there are no competing interests to declare.
